# The effect of age on long-term survival after pancreatic resection for pancreatic cancer

**DOI:** 10.1186/1471-2318-11-S1-A4

**Published:** 2011-08-24

**Authors:** Valentina Beltrame, Federico Tona, Margherita Moro, Carmelo Militello, Sergio Pedrazzoli, Claudio Pasquali, Cosimo Sperti

**Affiliations:** 1 Department of Surgery, Clinica Chirurgica IV, University of Padua, Italy; 2 Department of Surgical and Gastroenterological Sciences, University of Padua, Padova, Italy

## Background

The incidence of pancreatic cancer is increasing, and an increasing proportion of patients with pancreatic carcinoma is older than 75 years. After surgical resection remains the treatment of choice for pancreatic neoplasms, an increasing number of elderly patients are being referred for pancreatic resection. Although in the early 1990s there was rarely an indication for major pancreatic resection in the elderly because of the high postoperative complication rate, mortality and limited survival time [1], more recently an acceptable morbidity rate and late outcome in patients with advanced age have been reported [2,3]. This retrospective study analyzed the effect of age on the survival of patients who underwent resection for pancreatic cancer.

## Materials and methods

Data were collected on 212 consecutive patients who underwent pancreatic resection between January 2000 and July 2009 in our department, divided into two groups: group 1, patients under 75 years of age, and group 2, patients 75 years of age or older. The two groups were compared in terms of demographic features, comorbidities, and surgical procedures. All of the patients underwent standardized preoperative assessment of general medical conditions, blood tests, tumor marker CA 19-9 determination, abdominal CT scan, and when needed, magnetic resonance imaging or positron emission tomography. Surgical techniques included pylorus-preserving pancreaticoduodenectomy for tumors of the head, of the pancreas and distal splenopancreatectomy for tumors located in the body or tail. Total pancreatectomy was reserved for microscopic invasion of the line of resection. The morbidity and mortality rate included all complications or deaths after surgery until discharge from hospital. Age, stage, lymph node status, grading and radicality of resection were recorded as potentially prognostic factors. Statistical analysis was performed using the SPSS for Windows rel. 15.0. Patient overall survival and disease-free survival (DFS) were evaluated using the Kaplan-Meier method and compared with Log-Rank test. Independent prognostic variables were examined with Cox regression analysis. Statistical significance was considered as p< 0.05.

## Results

There were 107 males and 105 females. 49 patients were 75 years old or older (24 older than 80 years) and 163 were under 75 years of age. Clinicopathologic features of the two groups are detailed in Table 1. There were no significant differences regarding gender, type of operation, pathological findings, morbidity and mortality rate between the two age groups. The tumor’s grading and radicality of resection were independent prognostic factors both for disease-free survival, and overall survival. Age did not influence disease-free or overall survival in univariate or multivariate analysis. In the group of patients of an older age, disease-free survival was influenced by radicality of resection and the stage of the tumor, while overall survival was significantly impacted only by the stage of the tumor.

**Table 1:** Clinical findings of patients who underwent resection for pancreatic cancer.

	**<75** yrs (n=163)	**>75 yrs (**n=49**)**
**Gender**		
Male	88	19
Female	75	30
**Operation**		
Pancreaticoduodenectomy	119	37
Total pancreatectomy	5	2
Distal pancreatectomy	39	10
**Operative morbidity**	51(31.2%)	16(32.6%)
**Operative mortality**	3(1.8%)	1(2.0%)
**Median survival** (months)		
Disease-free	10	10
Overall	19	16

## Conclusions

The results of the present study strongly suggest that age should not be considered as a contraindication for major pancreatic resection in pancreatic cancer patients. Postoperative morbidity and mortality are not statistically different in the two age groups. Similarly, disease-free and overall survival of patients 75 years or older undergoing resection for pancreatic cancer are not substantially different from the survival expected in younger patients.

## References

[B1] Bathe OF Caldera H Hamilton KL Franceschi D Sleeman D Livingstone AS Levi JU Diminished benefit from resection of cancer of the head of the pancreas in patients of advanced age J Surg Oncol 2001 77 115 122 10.1002/jso.1081 11398165

[B2] Riall TA What is the effect of age on pancreatic resection? Adv Surg 2009 233 249 10.1016/j.yasu.2009.02.004 PMC2855222 19845182

[B3] Hardacre JM Simo K McGee MF Stellato TA Schulak JA Pancreatic resection in octogenarians J Surg Res 2009 156 129 132 10.1016/j.jss.2009.03.047 19592032

